# Impact of one versus two doses of mesenchymal stromal cells on lung and cardiovascular repair in experimental emphysema

**DOI:** 10.1186/s13287-018-1043-6

**Published:** 2018-11-08

**Authors:** Hananda A. Poggio, Mariana A. Antunes, Nazareth N. Rocha, Jamil Z. Kitoko, Marcelo M. Morales, Priscilla C. Olsen, Miquéias Lopes-Pacheco, Fernanda F. Cruz, Patricia R. M. Rocco

**Affiliations:** 1 Laboratory of Pulmonary Investigation, Carlos Chagas Filho Biophysics Institute, Federal University of Rio de Janeiro, Centro de Ciências da Saúde, Avenida Carlos Chagas Filho, 373, Bloco G1-014, Ilha do Fundão, Rio de Janeiro, Rio de Janeiro 21941-902 Brazil; 2 Department of Physiology and Pharmacology, Biomedical Institute, Fluminense Federal University, Niterói, Brazil; 3 Laboratory of Cellular and Molecular Physiology, Carlos Chagas Filho Biophysics Institute, Federal University of Rio de Janeiro, Rio de Janeiro, Brazil; 4 Laboratory of Clinical Bacteriology and Immunology, Faculty of Pharmacy, Federal University of Rio de Janeiro, Rio de Janeiro, Brazil; 5 National Institute of Science and Technology for Regenerative Medicine, Rio de Janeiro, Brazil

**Keywords:** Immunosuppression, Inflammation, Collagen fiber, Elastic fiber, Emphysema, Heart, Animal models

## Abstract

**Background:**

A single administration of mesenchymal stromal cells (MSCs) has been shown to reduce lung inflammation in experimental elastase-induced emphysema; however, effects were limited in terms of lung-tissue repair and cardiac function improvement. We hypothesized that two doses of MSCs could induce further lung and cardiovascular repair by mitigating inflammation and remodeling in a model of emphysema induced by multiple elastase instillations. We aimed to comparatively investigate the effects of one versus two doses of MSCs, administered 1 week apart, in a murine model of elastase-induced emphysema.

**Methods:**

C57BL/6 mice were randomly divided into control (CTRL) and emphysema (E) groups. Mice in the E group received porcine pancreatic elastase (0.2 IU, 50 μL) intratracheally once weekly for four consecutive weeks; the CTRL animals received sterile saline (50 μL) using the same protocol. Three hours after the last instillation, the E group was further randomized to receive either saline (SAL) or murine MSCs (10^5^ cells) intratracheally, in one or two doses (1 week apart). Fourteen days later, mice were euthanized, and all data analyzed.

**Results:**

Both one and two doses of MSCs improved lung mechanics, reducing keratinocyte-derived chemokine and transforming growth factor-β levels in lung homogenates, total cell and macrophage counts in bronchoalveolar lavage fluid (BALF), and collagen fiber content in airways and blood vessels, as well as increasing vascular endothelial growth factor in lung homogenates and elastic fiber content in lung parenchyma. However, only the two-dose group exhibited reductions in tumor necrosis factor-α in lung tissue, BALF neutrophil and lymphocyte count, thymus weight, and total cellularity, as well as CD8^+^ cell counts and cervical lymph node CD4^+^ and CD8^+^ T cell counts, as well as further increased elastic fiber content in the lung parenchyma and reduced severity of pulmonary arterial hypertension.

**Conclusions:**

Two doses of MSCs enhanced lung repair and improvement in cardiac function, while inducing T cell immunosuppression, mainly of CD8^+^ cells, in elastase-induced emphysema.

## Background

Emphysema is one of the phenotypes of chronic obstructive pulmonary disease (COPD), a global public health problem that affects an estimated 65 million people and causes more than 3 million deaths annually [1]. Rupture of alveolar walls, irreversible airspace enlargement, and inflammation, followed by a decline in lung function, are among the hallmarks of pulmonary emphysema [2, 3]. Moreover, extrapulmonary effects have also been described, such as cor pulmonale, skeletal muscle wasting, and weight loss, which significantly decrease quality of life and survival [1–4].

To date, there has been no effective therapy capable of modifying long-term decline in lung function in emphysema. Therefore, any new therapeutic strategy that could reduce airway inflammation and remodeling and mitigate extrapulmonary effects might represent a potential disease-modifying strategy. Several studies have demonstrated that mesenchymal stromal cells (MSCs) secrete paracrine factors that can modulate inflammation and remodeling in different experimental models of emphysema [5–9]. In a previous study conducted by our group, MSCs mitigated lung damage and improved cardiac function in experimental emphysema induced by multiple instillations of elastase; however, the effects were only modest in terms of lung-tissue repair [10]. Although a single dose of MSCs has been shown to reduce lung inflammation and fibrosis in experimental emphysema [6, 8, 10], more than one dose of MSCs may be required to induce efficient repair of lung and heart damage.

Within this context, we have undertaken the present study to comparatively investigate the effects of one versus two doses of MSCs, administered 1 week apart, in a murine model of elastase-induced emphysema. The effects on lung mechanics, histology, protein biomarkers in lung tissue, and cellularity in bronchoalveolar lavage fluid, bone marrow, thymus, and cervical and mediastinal lymph nodes, as well as cardiac function, were evaluated.

## Methods

The study protocol was approved by the Institutional Animal Care and Use Committee of the Health Sciences Center, Federal University of Rio de Janeiro, Brazil (chair: Prof. M. Frajblat; opinion no. 013/14). All animals received humane care in compliance with the “Principles of Laboratory Animal Care” formulated by the National Society for Medical Research and the US National Academy of Sciences *Guide for the Care and Use of Laboratory Animals*.

### Animal preparation and experimental protocol

Eighty female C57BL/6 mice (weight 20–25 g, age 8–10 weeks) were used for the experiment: 40 for evaluation of lung mechanics and histology and measurement of biomarker levels in lung tissue and 40 for analysis of total and differential cell counts in bronchoalveolar lavage fluid (BALF), bone marrow (BM), cervical (cLN) and mediastinal (mLN) lymph nodes, and thymus (*n* = 10/group). Animals were randomly assigned into two groups, control (CTRL) and emphysema (E), by the sealed-envelope method. Mice from group E (*n* = 60) received four intratracheal instillations of porcine pancreatic elastase (PPE, E1250, Sigma Chemical Co., St. Louis, MO, USA; 0.2 IU in 50 μL saline) at 1-week intervals (total dose, 0.8 IU PPE per animal). CTRL animals (*n* = 20) received saline alone (0.9% NaCl, 50 μL) using the same protocol [10, 11]. For all intratracheal instillations, mice were anesthetized with inhaled sevoflurane 2% (Sevorane®, Cristália, Itapira, SP, Brazil). A midline cervical incision (1 cm) was made to expose the trachea, and saline or PPE was instilled using a bent 27-gauge tuberculin needle. The cervical incision was closed with 5-0 silk suture, and the mice returned to their cages. All animals received tramadol 50 mg kg^−1^ intraperitoneally (i.p.) (Tramadon®, Cristália, Itapira, SP, Brazil) after the surgical procedure. Three hours after the last instillation, animals in group E were further randomized into three groups to receive one or two doses of bone marrow-derived MSCs (1 × 10^5^ in 50 μL saline) or sterile saline (SAL, 0.9% NaCl, 50 μL) intratracheally. All data were analyzed 14 days after the last instillation of saline or elastase. All analyses were performed in blind fashion.

### Isolation and characterization of MSCs

The MSCs used in the present study had been previously characterized as such [12] and kindly provided by Dr. Soraia Carvalho Abreu. Briefly, a pool of bone marrow cells was obtained from the femurs and tibias of 10 male C57BL/6 mice [13]. Bone marrow-derived MSCs were cultured in low-glucose Dulbecco’s modified Eagle medium supplemented with 1% L-glutamine, 1% antibiotics (10,000 IU/mL penicillin and 10,000 mg/mL streptomycin, GIBCO), and 10% fetal bovine serum (FBS, GIBCO). MSCs were grown under standard cell-culture conditions (37 °C, 5% CO_2_, humidified chamber). Only third-passage cells of less than 80% confluence were used in this study.

### Echocardiography

For echocardiographic assessment of cardiac function, the animals were anesthetized with inhaled 1.5% isoflurane (Cristália, São Paulo, Brazil), shaved over the precordial region, and examined with a Vevo 770 system (VisualSonics®, Toronto, ON, Canada) coupled to a 30-MHz transducer. Images were obtained from the parasternal short-axis and long-axis views. B-dimensional parasternal short-axis views of both ventricles were acquired at the level of the papillary muscles for calculation of left and right ventricular areas. Pulsed-wave Doppler was used to measure pulmonary artery acceleration time (PAT) and pulmonary artery ejection time (PET), from which their ratio (PAT/PET), an indirect marker of pulmonary arterial hypertension, was derived [10, 11, 14]. The diastolic dimension of the right ventricle was also measured. All parameters were acquired in accordance with American Society of Echocardiography and European Association of Cardiovascular Imaging recommendations [15].

### Mechanical parameters

Fourteen days after the last administration of saline or PPE, animals from the CTRL and E groups were premedicated with diazepam 10 mg kg^−1^ i.p. (Compaz®, Cristália, Itapira, SP, Brazil), anesthetized with thiopental sodium 20 mg kg^−1^ i.p. (Thiopentax®, Cristália, Itapira, SP, Brazil), tracheotomized, paralyzed with vecuronium bromide 0.005 mg kg^−1^ i.v. (Vecuron®, Cristália, Itapira, SP, Brazil), and ventilated with a constant-flow ventilator (Samay VR15; Universidad de la Republica, Montevideo, Uruguay) set to a respiratory rate of 100 bpm, tidal volume (*V*_T_) of 0.2 mL, and fraction of inspired oxygen (FiO_2_) of 0.21. The anterior chest wall was surgically removed and a positive end-expiratory pressure (PEEP) of 2 cmH_2_O applied. Airflow and tracheal pressure (Ptr) were measured. In an open-chest preparation, Ptr reflects transpulmonary pressure (PL). Lung mechanics were analyzed by the end-inflation occlusion method [10, 14, 16, 17]. Static lung elastance (Est,L) was determined by dividing lung elastic recoil pressure (Pel) by V_T_. Est,L was measured 12 times in each animal. All data were analyzed in ANADAT software (RHT-InfoData Inc., Montreal, Quebec, Canada). All experiments lasted less than 15 min.

### Lung histology

Immediately after acquisition of lung mechanics parameters, laparotomy was performed and 1000 IU of heparin (Hemofol®, Cristália, Itapira, SP, Brazil) injected into the vena cava. The trachea was clamped at end-expiration (PEEP = 2 cmH_2_O) in all groups to avoid distortion of lung morphometry [10, 11, 14, 18]. Mice were killed by exsanguination following transection of the abdominal aorta and vena cava. The left lung was then removed, fixed in 10% buffered formalin, and embedded in paraffin. Slices (4-μm-thick) were cut and stained with hematoxylin–eosin. Lung histology analysis was performed with an integrating eyepiece with a coherent system consisting of a grid with 100 points and 50 lines of known length coupled to a conventional light microscope (Olympus BX51, Olympus Latin America Inc., Brazil). The volume fractions of the lung occupied by collapsed alveoli (alveoli with rough or plicate walls), normal pulmonary areas, or hyperinflated structures (alveolar ducts, alveolar sacs, or alveoli, all with maximal chord length in air > 120 mm) were determined by the point-counting technique [19] across 10 random, non-coincident microscopic fields. Briefly, points falling on collapsed lung, normal pulmonary areas, or hyperinflated structures were counted and divided by the total number of points in each microscopic field. Enlargement of air spaces was evaluated by measurement of mean linear intercept (Lm) [10, 11, 14, 20]. Total cell, neutrophil, and mononuclear cell counts were also determined by the point-counting technique across 10 random, non-coincident microscopic fields, at × 1000 magnification.

The collagen fiber content was computed in airways and pulmonary vessel walls by the Picrosirius polarization method. Elastic fiber content was computed in alveolar septa using Weigert’s resorcin–fuchsin method with oxidation. Images were generated by a microscope (Axioplan, Zeiss) connected to a digital camera (Sony Trinitron CCD, Sony, Tokyo, Japan) and fed into a computer through a frame grabber (Oculus TCX, Coreco, St Laurent, QC, Canada) for offline processing. The thresholds for collagen and elastic fibers were established after enhancement of contrast up to the point in which fibers were easily identified as either birefringent (collagen) or black (elastic) bands at × 400 magnification, in ImagePro Plus 7.1 Software (Media Cybernetics, Silver Spring, MD, USA) [10, 11, 18]. The areas occupied by the elastic and collagen fibers were measured by digital densitometric recognition, divided by the tissue of each studied area, and expressed as the percentage of elastic fiber in the alveolar septa and collagen fiber in the airways or pulmonary vessel wall.

### Total and differential cell counts

Bronchoalveolar lavage fluid (BALF) was obtained by gentle aspiration of 400 μL of PBS 1× injected into the airways three times (final volume 1.2 mL) via a tracheal cannula. BALF was centrifuged at 250*g* for 10 min at 4 °C. Cell pellets were resuspended in PBS 1×.

Bone marrow (BM) was obtained by gentle lavage of the right femur of each animal with 1 mL of PBS 1×.

Cervical and mediastinal lymph nodes (cLN and mLN, respectively) were carefully extracted and placed in 1 mL of PBS 1×. Cell suspensions were obtained after mechanical homogenization.

The thymus of each animal was carefully extracted and placed in 1 mL of PBS 1×. Again, cell suspensions were obtained after mechanical homogenization.

Total leukocytes from BALF, BM, cLN, mLN, and thymus were obtained as previously described [21] and counted in a Neubauer chamber after dilution with Turk’s solution (2% acetic acid). Thereafter, BALF and BM cells were pelleted onto glass slides by cytocentrifugation and stained by the May-Grünwald-Giemsa method for differential cell counts as described elsewhere [12, 21].

Cell populations from the BALF, cLN, mLN, and thymus were characterized using a FACSCalibur flow cytometer (Becton Dickinson Biosciences, San Jose, CA, USA) after incubation with anti-CD4, CD8, CD25, and Foxp3 antibodies (eBiosciences, San Diego, CA, USA), as described [22]. Analyses were performed in FlowJo software version 10.0.7 (Tree Star Inc., Ashland, OR, USA).

### Enzyme-linked immunosorbent assay (ELISA)

Protein levels of keratinocyte-derived chemokine (KC, a mouse analog of interleukin [IL]-8), tumor necrosis factor (TNF)-α, IL-10, vascular endothelial growth factor (VEGF), and transforming growth factor (TGF)-β in lung homogenate were evaluated by ELISA using matched antibodies from PeproTech (Rocky Hill, NJ, USA) and R&D Systems (Minneapolis, MN, USA), in accordance with manufacturer instructions. Total protein content was quantified by Bradford’s reagent (Sigma-Aldrich, St Louis, MO, USA). The concentration (pg/mL) was normalized to total protein content (pg/mg total protein).

### Statistical analysis

Sample size calculation was based on pilot studies and on previous studies in a murine model of elastase-induced emphysema conducted in our laboratory [10, 11, 14, 16, 18, 20]. A sample size of 10 animals per group would provide the appropriate power (1 − *β* = 0.8) to identify significant (*α* = 0.05) differences in mean linear intercept between CTRL and E groups, taking into account an effect size *d* = 1.97, a two-sided test, and a sample size ratio of 1 (G *- Power 3.1.9.2, University of Düsseldorf, Germany).

Data were tested for normality using the Kolmogorov–Smirnov test with Lilliefors’ correction, while the Levene median test was used to evaluate homogeneity of variances. If both conditions were satisfied, differences among groups were assessed using one-way analysis of variance (ANOVA) followed by Tukey’s test. For nonparametric results, the Kruskal–Wallis test followed by Dunn’s test was used.

Parametric data were expressed as mean ± SD, while nonparametric data were expressed as median (interquartile range). All tests were performed in GraphPad Prism v6.07 (GraphPad Software, La Jolla, California, USA). Significance was established at *p* < 0.05.

## Results

### Administration of one and two doses of MSCs modulated protein levels of mediators, but a two-dose regimen further mitigated inflammation and morphological changes in the lungs

Initially, we investigated the impact of one vs. two doses of MSC-based therapy on inflammation in the lung, as it is the primary tissue injured in emphysema [3, 5, 10].

**Fig. 1:**
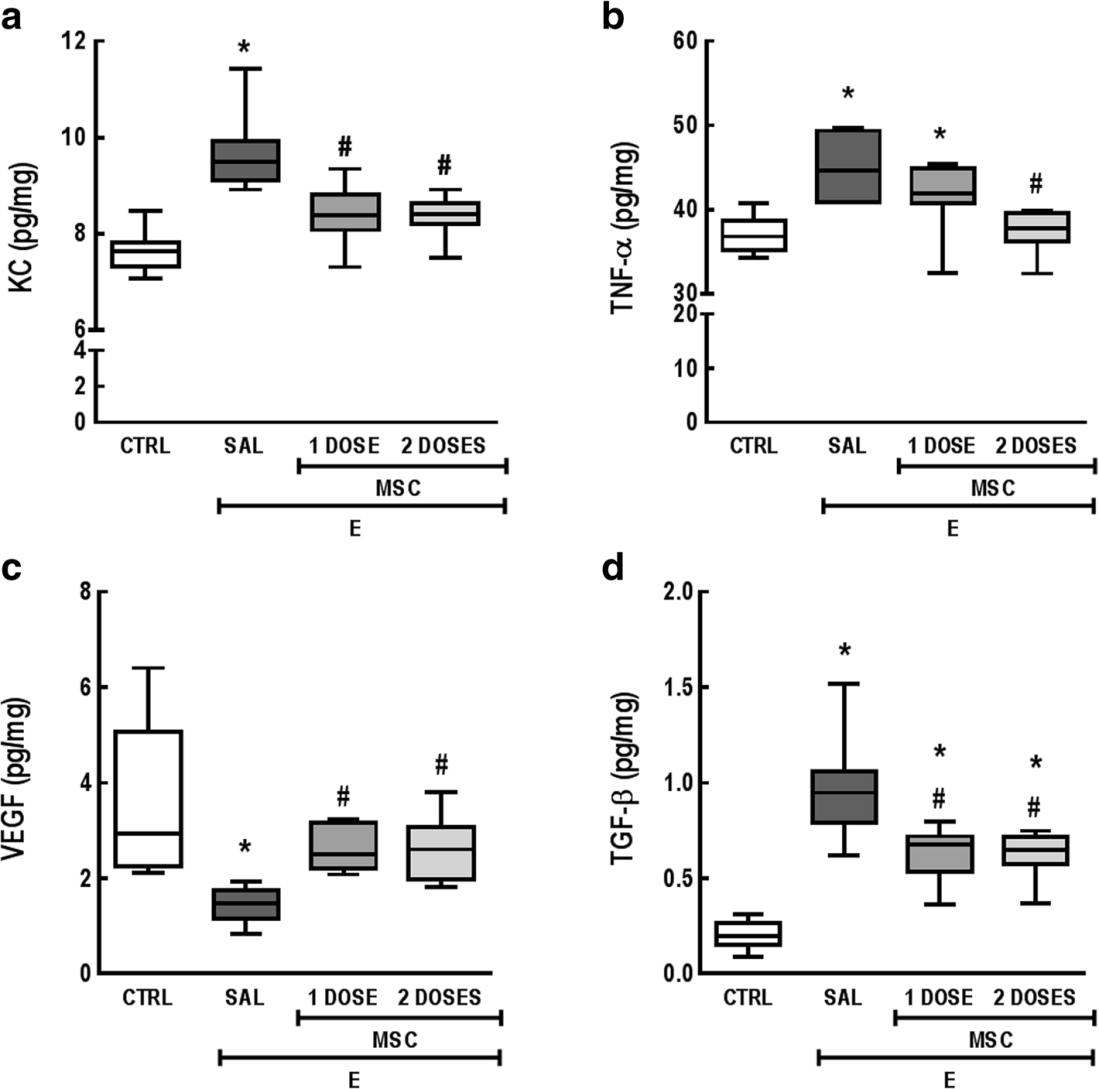
MSC administration modulated protein levels of relevant mediators. Protein levels of **a** keratinocyte-derived chemokine (KC, a mouse analog of interleukin [IL]-8), **b** tumor necrosis factor (TNF)-α, **c** vascular endothelial growth factor (VEGF), and **d** transforming growth factor (TGF)-β in lung homogenate tissue. CTRL control mice, E emphysema mice, SAL saline-treated mice, MSC MSC-treated group. The Kruskal–Wallis test followed by Dunn’s test was used for statistical comparison. Data are presented as median ± interquartile range. *N* = 10 animals/group. *Significantly different from CTRL (*p* < 0.05). ^#^Significantly different from E-SAL (*p* < 0.05)

The E-SAL group exhibited higher protein levels of KC, TNF-α, and TGF-β, while VEGF levels were significantly reduced compared to the CTRL group. Either one or two doses of MSCs reduced KC and TGF-β levels, while increasing VEGF levels compared to E-SAL. However, only the two-dose group exhibited significant reductions in TNF-α levels compared to E-SAL animals (Fig. 1a–d). No significant differences were observed in IL-10 levels among the groups (data not shown).

The E-SAL group also exhibited greater fractions of collapsed and hyperinflated areas, Lm, as well as higher neutrophil and mononuclear cell counts in lung tissue compared to the CTRL group. Either one or two doses of MSCs mitigated these parameters; however, two doses were more effective at mitigating hyperinflated areas, Lm, and neutrophil cell count (Table 1).

**Table 1:** Lung morphometry

			Normal (%)	Collapse (%)	Hyperinflation (%)	Lm (μm)	Neutrophils (%)	Mononuclear cells (%)
CTRL			91.7 ± 2.1	8.3 ± 2.1	0.0 ± 0.0	22.3 ± 5.1	1.6 ± 0.7	33.8 ± 2.3
E	SAL		18.4 ± 4.9*	27.2 ± 8.6*	54.4 ± 9.9*	50.3 ± 6.7*	7.3 ± 0.8*	42.4 ± 3.6*
	MSC	1 dose	41.5 ± 4.8*^,#^	15.9 ± 4.8*^,#^	42.6 ± 4.1*^,#^	33.4 ± 2.8*^,#^	2.9 ± 0.9*^,#^	31.6 ± 4.0^#^
		2 doses	51.4 ± 11.3*^,#,ǂ^	17.9 ± 4.4*^,#^	30.7 ± 6.3*^,#,ǂ^	29.8 ± 4.2*^,#,ǂ^	1.7 ± 0.5^#,ǂ^	36.1 ± 3.3^#^

Volume fraction of normal, collapsed, and hyperinflated pulmonary areas, mean linear intercept (Lm), and neutrophil and mononuclear cell counts in lung tissue. One-way ANOVA followed by Tukey’s test was used for statistical comparison. Data are presented as means ± SD. *N* = 10 animals/group

*CTRL* control mice, *E* emphysema mice, *SAL* saline-treated mice, *MSC* MSC-treated mice

*Significantly different from CTRL (*p* < 0.05)

^#^Significantly different from E-SAL (*p* < 0.05)

^ǂ^Significantly different from E-MSC-1 dose (*p* < 0.05)

**Fig. 2:**
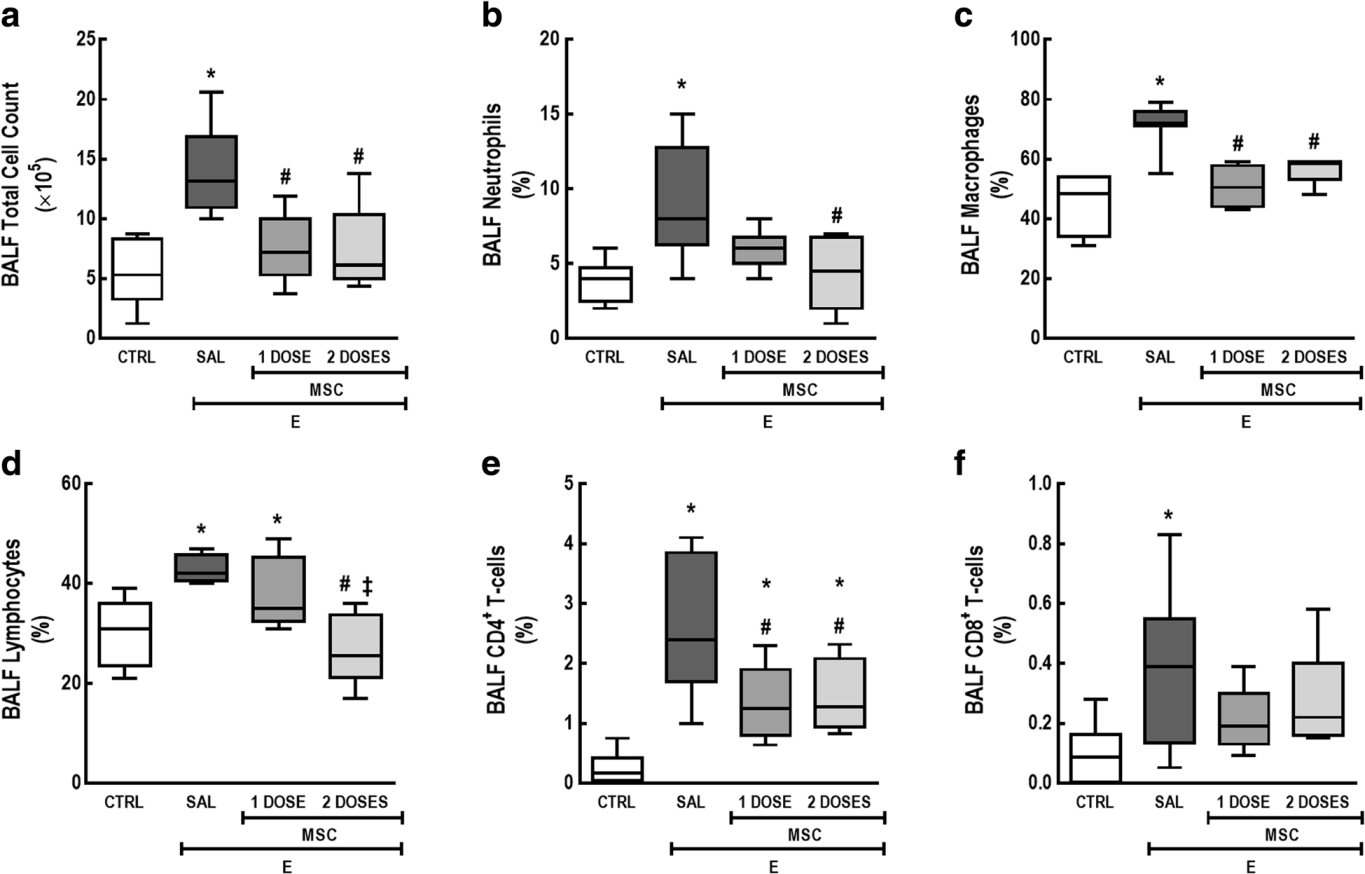
Two doses of MSCs led to further mitigation in BALF cellularity compared to a one-dose regimen. **a** Total leukocytes, **b** neutrophils, **c** macrophages, **d** lymphocytes, **e** CD4^+^ T cells, and **f** CD8^+^ T cell counts in BALF. CTRL control mice, E emphysema mice, SAL saline-treated mice, MSC MSC-treated group, MSCs mesenchymal stromal cells, BALF bronchoalveolar lavage fluid. One-way ANOVA followed by Tukey’s test was used for statistical comparison. Data are presented as means ± SD. *N* = 10 animals/group. *Significantly different from CTRL (*p* < 0.05). ^#^Significantly different from E-SAL (*p* < 0.05)

In the BALF, E-SAL animals exhibited an increased total cell count as well as a greater percentage of neutrophils, macrophages, lymphocytes, CD4^+^ T cells, and CD8^+^ T cells compared to CTRL. Either one or two doses of MSCs reduced BALF total cell count, as well as percentages of macrophages and CD4^+^ T cell. However, only two doses of MSCs were able to significantly reduce neutrophil and lymphocyte counts compared to E-SAL. Neither one nor two doses of MSCs reduced CD8^+^ T cell percentages compared to the E-SAL group (Fig. 2a–f).

### Two doses of MSCs led to further mitigation of inflammation in lymphoid tissues

To evaluate the effects of one or two doses of MSC-based therapy on systemic inflammation, we measured total and differential cell counts in BM and lymphoid tissues. In particular, percentages of CD4^+^ and CD8^+^ T lymphocytes as well as CD4^+^CD25^+^Foxp3^+^ (i.e., Treg) cells were investigated, since emphysema can lead to an imbalance in lymphocyte subpopulations [23–25].

**Fig. 3:**
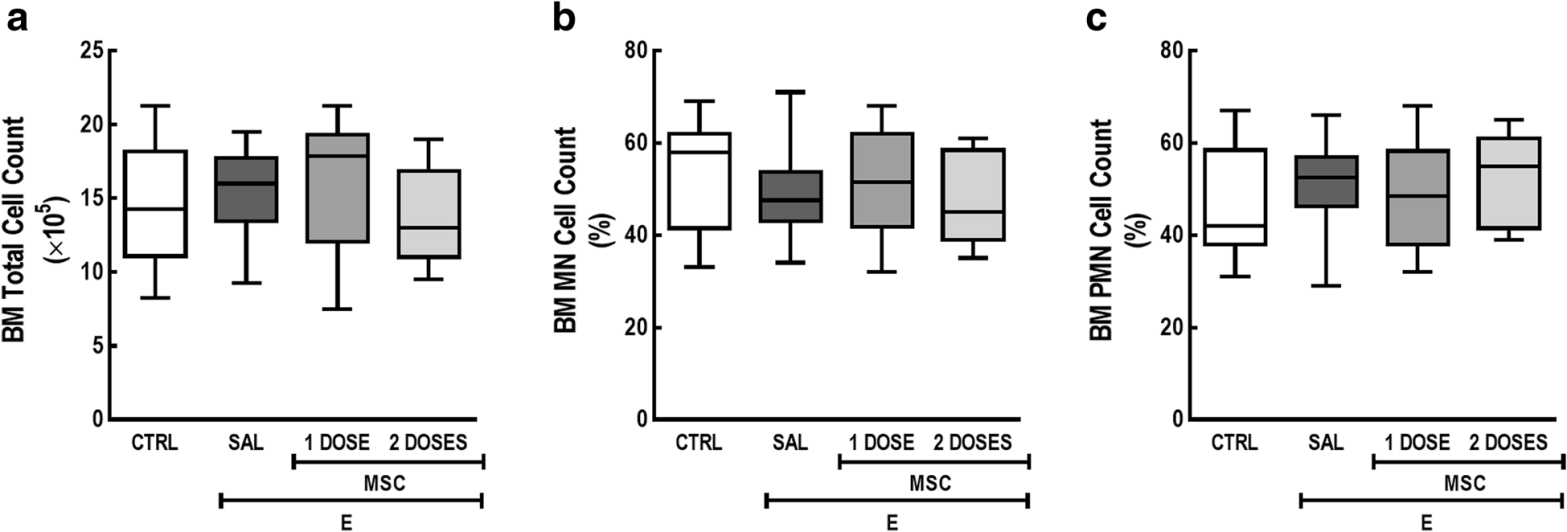
MSC administration did not affect BM cellularity, regardless of dose regimen. **a** Total leukocytes, **b** MN, and **c** PMN cell counts in BM. CTRL control mice, E emphysema mice, SAL saline-treated mice, MSC MSC-treated group, MSCs mesenchymal stromal cells, BM bone marrow, MN mononuclear cells, PMN polymorphonuclear cells. One-way ANOVA followed by Tukey’s test was used for statistical comparison. Data are presented as means ± SD. *N* = 10 animals/group

In the BM, no significant differences in total and differential cell count were observed among groups (Fig. 3a–c).

**Fig. 4:**
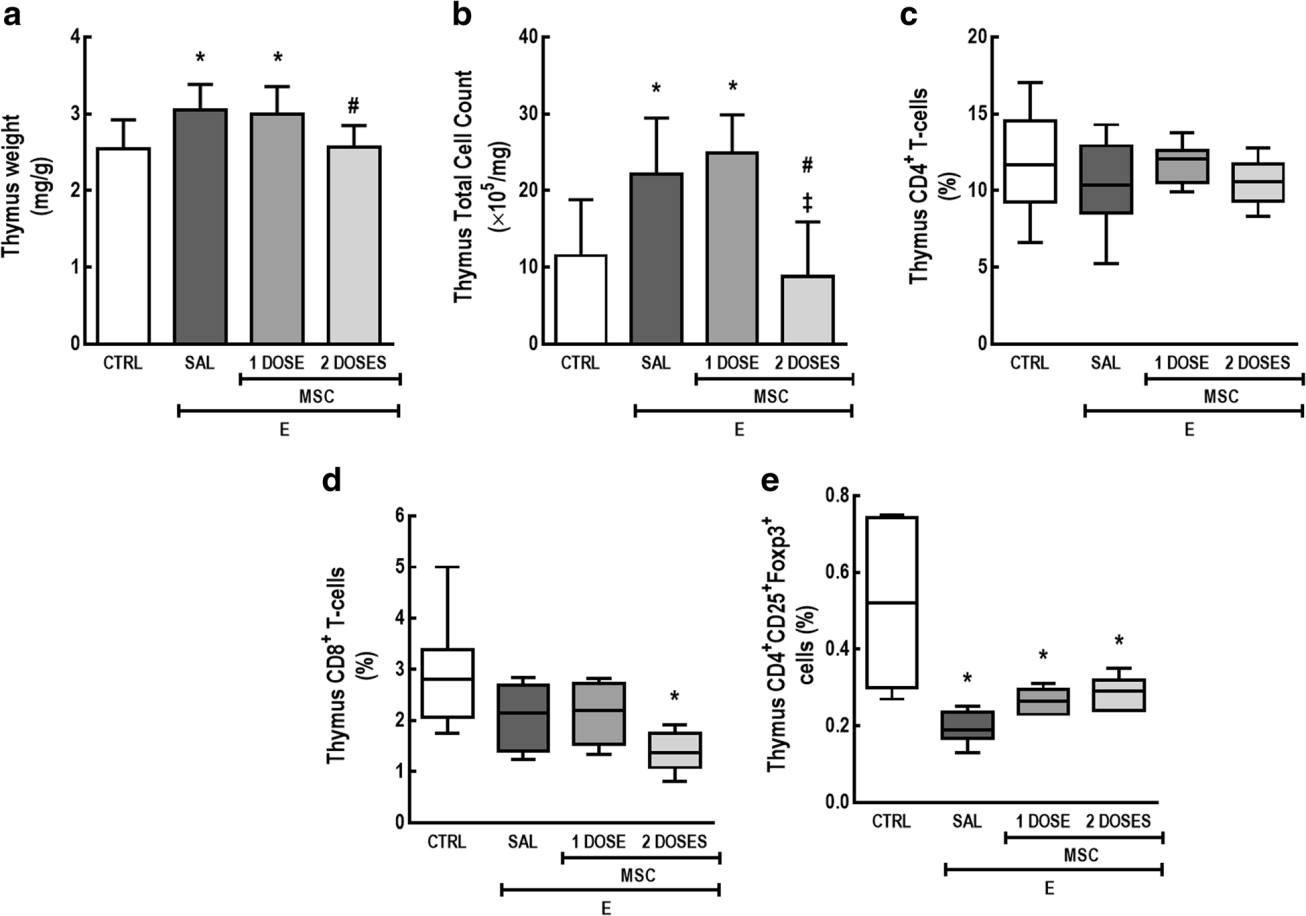
Two doses of MSCs, but not a one-dose regimen, led to immunosuppression in the thymus. **a** Thymus weight, **b** total cell count, **c** CD4^+^ T cells, **d** CD8^+^ T cells, and **e** CD4^+^CD25^+^Foxp3^+^ cell counts in thymus. CTRL control mice, E emphysema mice, SAL saline-treated mice, MSC MSC-treated group, MSCs mesenchymal stromal cells. For statistical comparison, the following tests were used: one-way ANOVA followed by Tukey’s test in panels **a**, **b** and Kruskal–Wallis test followed by Dunn’s test in panels **c**–**e**. Data are presented as means ± SD (**a**, **b**) or median ± interquartile range (**c**–**e**). *N* = 10 animals/group. *Significantly different from CTRL (*p* < 0.05). ^#^Significantly different from E-SAL (*p* < 0.05). ^‡^Significantly different from E-MSC-1 dose (*p* < 0.05)

In the thymus, the E-SAL group exhibited greater weight and total cell count compared to CTRL mice. Two doses of MSCs, but not one dose regimen, reduced thymus weight and total cell count compared to E-SAL. In addition, two doses of MSCs significantly reduced the percentage of CD8^+^ T cells compared to CTRL. The E-SAL group also exhibited a reduction in thymus CD4^+^CD25^+^Foxp3^+^ cell count compared to CTRL mice, whereas neither one nor two doses of MSCs were able to reverse this abnormality (Fig. 4a–e).

**Fig. 5:**
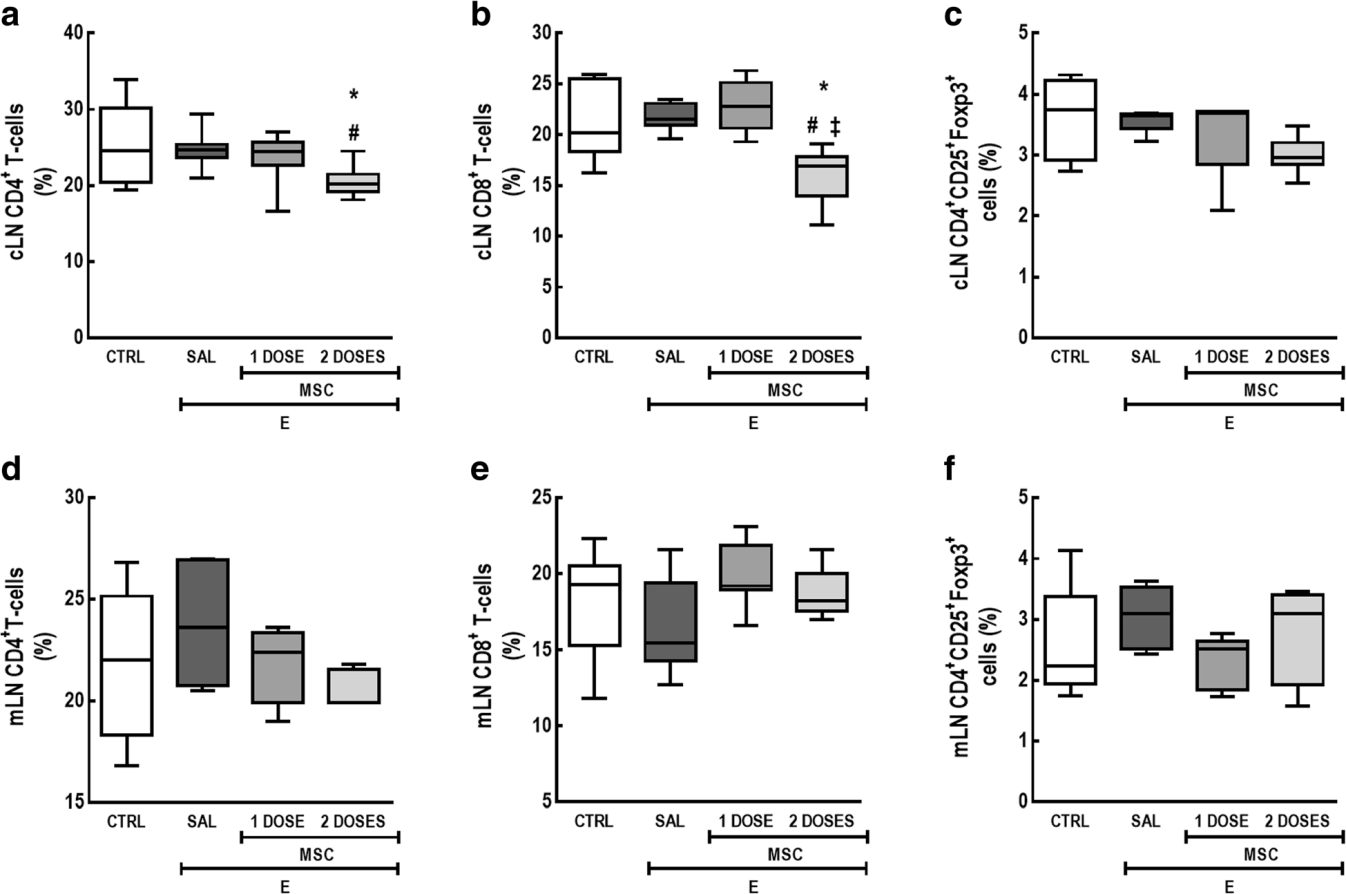
Two doses of MSCs led to immunosuppression in cervical lymph nodes, but not in mediastinal lymph nodes. **a** CD4^+^ T cells, **b** CD8^+^ T cells, and **c** CD4^+^CD25^+^Foxp3^+^ cell count in cLN; **d** CD4^+^ T cells, **e** CD8^+^ T cells, and **f** CD4^+^CD25^+^Foxp3^+^ cell count in mLN. CTRL control mice, E emphysema mice, SAL saline-treated mice, MSC MSC-treated group, MSCs mesenchymal stromal cells, cLN cervical lymph nodes, mLN mediastinal lymph nodes. The Kruskal–Wallis test followed by Dunn’s test was used for statistical comparison. Data are presented as median ± interquartile range. *N* = 10 animals/group. *Significantly different from CTRL (*p* < 0.05). ^#^Significantly different from E-SAL (*p* < 0.05). ^‡^Significantly different from E-MSC-1 dose (*p* < 0.05)

In the cLN, the CTRL and E-SAL groups exhibited similar percentages of CD4^+^, CD8^+^, and CD4^+^CD25^+^Foxp3^+^ cells (Fig. 5a–c). Two doses of MSCs, but not one-dose regimen, decreased the relative CD4^+^ and CD8^+^ T cell counts. In the mLN, no differences were observed in CD4^+^, CD8^+^, or CD4^+^CD25^+^Foxp3^+^ cell counts among the groups (Fig. 5d–f).

### One or two doses of MSCs similarly decreased collagen fiber content, but two doses of MSCs led to further elastogenesis

To further investigate the impact of one or two doses of MSC-based therapy, we analyzed effects on the remodeling process, as emphysema leads to loss of elastic fiber content and damaged tissue is replaced by collagen [10, 11, 16].

**Fig. 6:**
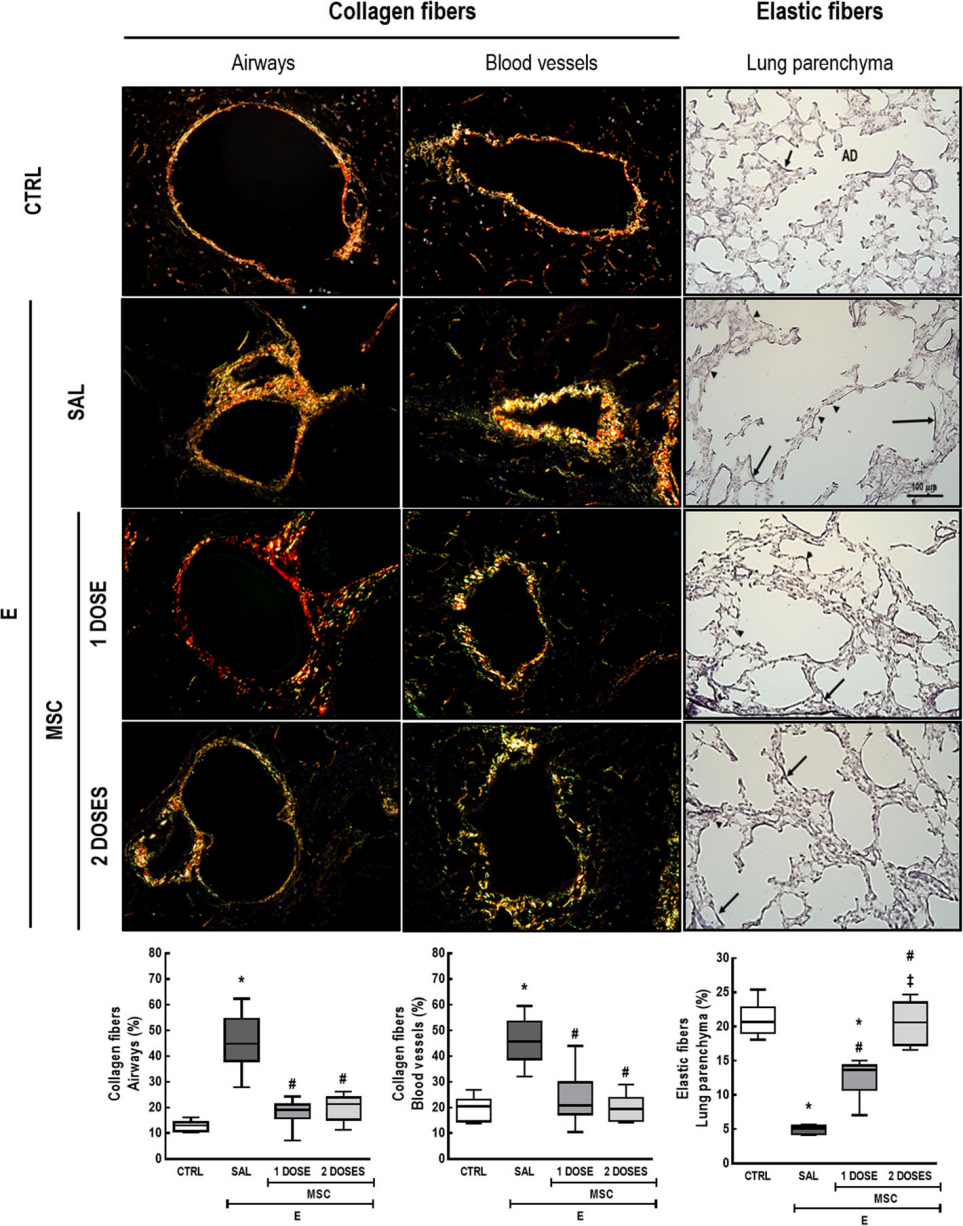
One or two doses of MSCs similarly decreased collagen fiber content, but two doses led to further elastogenesis. Collagen fiber content in airways and blood vessels and elastic fiber content on lung parenchyma. Arrows indicate elastic fibers (stained black). CTRL control mice, E emphysema mice, SAL saline-treated mice, MSC MSC-treated group, MSCs mesenchymal stromal cells. The Kruskal–Wallis test followed by Dunn’s test was used for statistical comparison. Data are presented as median ± interquartile range. *N* = 10 animals/group. *Significantly different from CTRL (*p* < 0.05). ^#^Significantly different from E-SAL (*p* < 0.05). ^‡^Significantly different from E-MSC-1 dose (*p* < 0.05)

Compared to the CTRL group, E-SAL mice exhibited higher collagen fiber content in the airways and blood vessels, while elastic fiber content was significantly reduced in the lung parenchyma. Either one or two doses of MSCs reduced collagen fiber content in the airways and blood vessels in emphysematous mice to CTRL-comparable levels. Elastic fiber content in lung parenchyma increased after one or two doses of MSCs; however, the effects were more pronounced after two doses (Fig. 6).

### One or two doses of MSC similarly improved lung mechanics, but only the two-dose regimen led to improvement in cardiac function

Finally, we investigated whether the beneficial effects of MSC-based therapy translated into improvements in cardiorespiratory function in experimental emphysema.

**Fig. 7:**
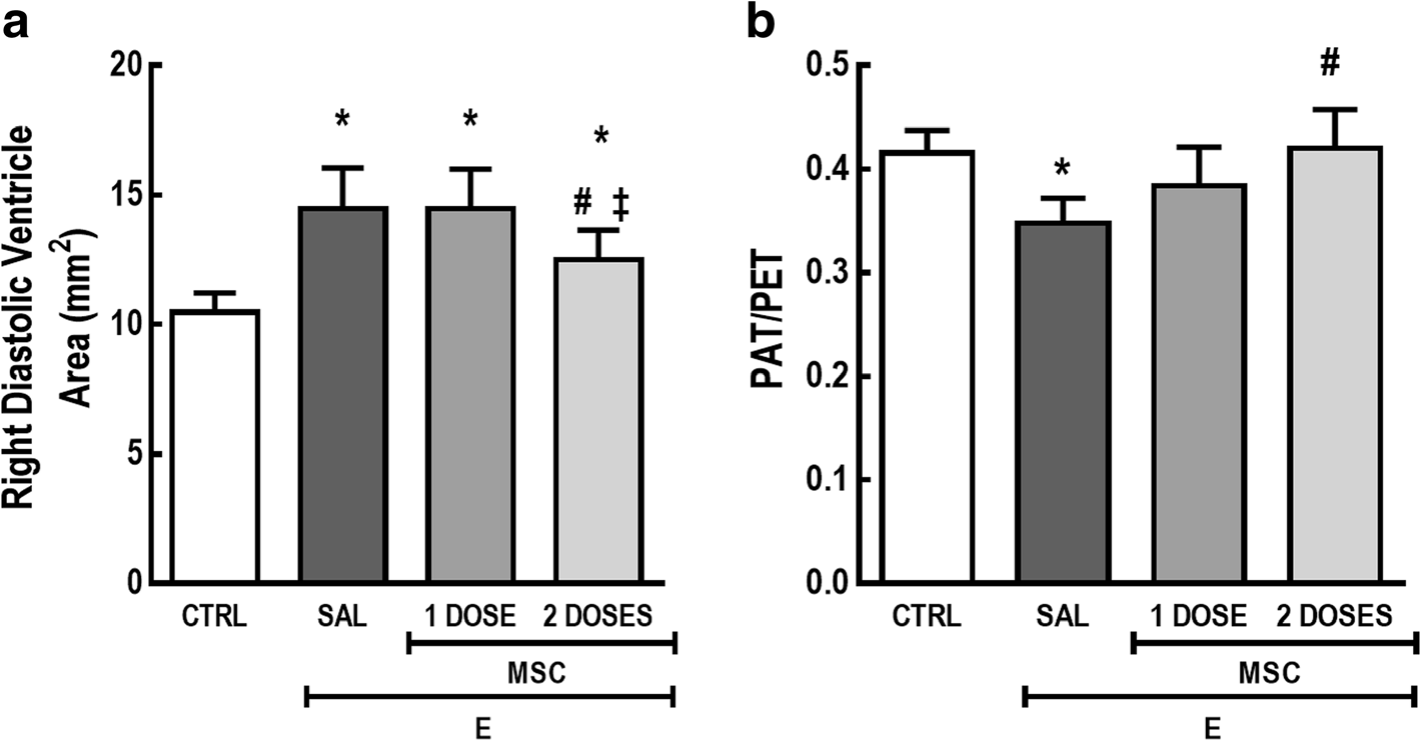
One or two doses of MSCs similarly improved lung mechanics. CTRL control mice, E emphysema mice, SAL saline-treated mice, MSC MSC-treated group, MSCs mesenchymal stromal cells, Est,L static lung elastance. One-way ANOVA followed by Tukey’s test was used for statistical comparison. Data are presented as means ± SD. *N* = 10 animals/group. *Significantly different from CTRL (*p* < 0.05). ^#^Significantly different from E-SAL (*p* < 0.05)

Est,L was reduced in the E-SAL group compared to CTRL animals. Either one or two doses of MSCs enhanced Est,L of emphysematous mice (Fig. 7).

**Fig. 8:**
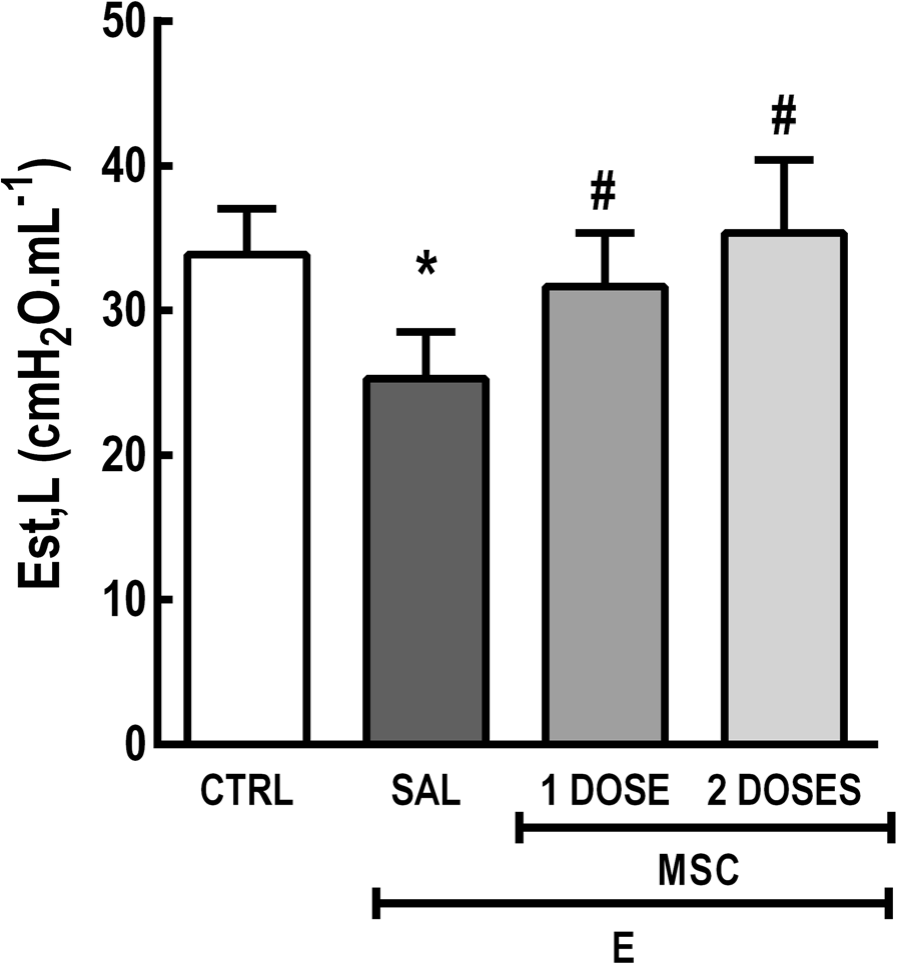
Two doses of MSCs, but not a one-dose regimen, led to improvement in cardiac function. **a** Diastolic right ventricle area and **b** PAT/PET ratio. CTRL control mice, E emphysema mice, SAL saline-treated mice, MSC MSC-treated group, MSCs mesenchymal stromal cells, PAT pulmonary artery acceleration time, PET pulmonary artery ejection time. One-way ANOVA followed by Tukey’s test was used for statistical comparison. Data are presented as means ± SD. *N* = 10 animals/group. *Significantly different from CTRL (*p* < 0.05). ^#^Significantly different from E-SAL (*p* < 0.05). ^‡^Significantly different from E-MSC-1 dose (*p* < 0.05)

E-SAL mice also exhibited an increase in the diastolic right ventricle area, while PAT and PET ratio were reduced compared to the CTRL group. Only two doses of MSCs were able to significantly mitigate such abnormalities (Fig. 8).

## Discussion

In the model of elastase-induced emphysema used herein, two doses of MSCs, compared to a single dose, yielded further decrease levels of TNF-α in lung tissue, fractional area of hyperinflation in the lung, Lm, neutrophil cell counts in lung tissue, BALF neutrophil and lymphocyte cell counts, thymus total and CD8^+^ cell counts, and cLN CD4^+^ and CD8^+^ T cell counts, while increasing elastic fiber content in the lung parenchyma and reducing the diastolic right ventricle area.

The use of porcine pancreatic elastase (PPE) to induce emphysema experimentally is more advantageous than cigarette smoke exposure, as PPE is inexpensive and able to induce greater and more widespread lung damage [26, 27]. In addition, lung damage induced by elastase persists for longer after induction, in contrast to cigarette smoke [28].

In the present study, we used a model of multiple instillations of elastase [10, 11, 14, 16, 18, 20]. This model results in persistent lung inflammation, elastolysis, and airway and vessel collagen deposition, as well as cardiac impairment [27], thereby more closely resembling human emphysema. MSCs were administered 3 h after the last elastase challenge, when lung-tissue damage and cardiac impairment were already established [16]. Our design is thus in contrast with that of previous reports in which cell-based therapy was administered prophylactically [20, 29]. In addition, MSCs were harvested from BM, since cells from this source have been shown to yield greater therapeutic effects, including macrophage polarization toward the anti-inflammatory M2 profile, in comparison to cells obtained from adipose and lung tissues [10]. MSCs were administered intratracheally, since this route led to a greater reduction in alveolar hyperinflation than systemic delivery in previous studies [10]. A 1-week interval between treatments was chosen since we observed that, at this time point, one dose had yielded beneficial effects on lung inflammation and fibrosis [10]; therefore, a second dose might produce further improvement in these parameters.

MSCs can secrete and stimulate resident cells to secrete multiple paracrine factors that induce immunomodulatory effects on the injured environment [12, 13, 30–32]. Previous studies have shown that a single dose of MSCs, whether administered locally or systemically, mitigated lung inflammation in experimental emphysema [6, 8–10, 33, 34]. In the present study, both one and two doses of MSCs reduced lung-tissue levels of KC, an important biomarker involved in the pathophysiology of emphysema. This chemokine is produced by macrophages and attracts neutrophils to the lungs, leading to further tissue damage [3, 35]. Thus, the reduction in KC levels observed after MSC-based therapy can be correlated to a decreased macrophage count in the lungs.

Neutrophil recruitment into lung tissue contributes to disease development and progression because these cells release several proteases, including neutrophil elastase, that lead to elastolysis and airspace enlargement [36–38]. In our model, elastase-induced emphysema significantly increased neutrophil counts in BALF and lung tissue as well as Lm and fractional hyperinflation, while reducing elastic fiber content in the lungs. Either one or two doses of MSCs reduced Lm and hyperinflation areas, as well as enhanced elastic fiber content; however, two doses of MSCs were more effective at reversing these parameters, which can be correlated to a significant reduction in neutrophilia and TNF-α levels [37, 38].

Lymphocytes also participate in disease development and progression, either by direct cytolytic activity or by secreting pro-inflammatory mediators [24, 39]. Lymphocyte traffic between lymphoid organs and lung tissue can stimulate proliferation, differentiation, and migration of other lymphocytes [40]. Furthermore, patients with COPD exhibit an imbalance in lymphocyte subpopulations, with increased CD4^+^ and CD8^+^ T cell and decreased Treg cell counts [23–25]. In particular, CD8^+^ T cells may mediate TNF-induced lung-tissue destruction by apoptotic mechanisms [23, 41]. In our emphysema model, both one and two doses of MSCs mitigated BALF CD4^+^ T cell count; however, only the two-dose regimen showed a reduction in percentage of total lymphocytes. In addition, emphysematous animals exhibited an increase in thymus weight and total cell count, which could be correlated with the migration of undifferentiated lymphocytes to the thymus, where they mature into T cells [39]. Only the two-dose regimen reduced thymus weight and total cell count as well as CD8^+^ T cell count. Two doses of MSCs also reduced CD4^+^ and CD8^+^ T cell counts in cLN. In fact, MSCs may induce immunosuppression in lymphoid tissues by arresting immature T cells in the G_0_/G_1_ phase of the cell cycle [42–44] and, thus, not affect those activated cells already present in inflammatory foci [45].

TGF-β plays an important role in the remodeling process of emphysema by stimulating fibroblast proliferation and secretion of collagen fibers [46], which is in line with the increased fibrosis in airways and blood vessels observed in our model. Either one or two doses of MSCs reduced collagen fiber content in airways and blood vessels to control-like levels. Nonetheless, only two doses of MSCs were able to completely restore elastic fiber content in the lung parenchyma. As inflammation, apoptosis, and oxidative stress may persist and continue to contribute to disease progression even after smoking cessation [3, 5], repeated administration of MSCs may be required to induce continued stimulation for tissue repair. In fact, repeated administration of BM cells was also needed to prevent disease progression in a model of silica-induced lung fibrosis [47].

As the disease progresses, COPD patients may develop impairment of cardiovascular function [2, 4]. An increase in neutrophil elastase has been correlated with VEGF fragmentation, leading to loss of crucial signaling for endothelial-cell survival [36, 48]. Consequently, remodeling of vascular lung structure results in right ventricular overload [14, 48, 49]. In the present study, either one or two doses of MSCs enhanced VEGF levels as well as reduced collagen fiber content in blood vessels, which is consistent with previous in vitro and in vivo reports [10, 20, 50, 51]. However, only the two-dose regimen was able to reverse changes in PAT/PET ratio and diastolic right ventricle dimension, which suggests that repeated administration of MSCs may be required to progressively reverse pulmonary arterial hypertension when it is already established.

In previous studies of experimental emphysema performed by our group, mice demonstrated a mild-to-moderate disease severity in which cardiac function was compromised as well as lung morphology and ultrastructure were damaged, but they did not exhibit impairment in lung function [10, 20]. In the present study, severe disease was induced by increasing elastase doses (0.1 vs. 0.2 IU), which successfully led to reduction of Est,L in emphysematous mice. Although other reports using different models of emphysema have shown a mismatch between the degree of tissue loss and the severity of pulmonary dysfunction [52–54], the improvement in lung mechanics after MSC administration may be attributed to tissue repair and re-establishment of lung structure. In this line, both one and two doses of MSCs reduced Lm, lung collapse, lung hyperinflation, inflammatory cell counts, and airway fibrosis, while increasing elastic fiber content in the lung parenchyma. Altogether, this resulted in significant improvement of lung function.

## Conclusions

Compared to a single dose of BM-derived MSCs, two doses of MSCs yielded further lung repair and improvement in cardiac function in elastase-induced emphysema. Additionally, the two-dose regimen was associated with T cell immunosuppression, mainly of CD8^+^ cells.

## Abbreviations

ANOVA: Analysis of variance
BALF: Bronchoalveolar lavage fluid
BM: Bone marrow
CTRL: Control
E: Emphysema
ELISA: Enzyme-linked immunosorbent assay
KC: Keratinocyte-derived chemokine
MN: Mononuclear cells
MSC: Mesenchymal stromal cells
PAT: Pulmonary artery acceleration time
PEEP: Positive end-expiratory pressure
PET: Pulmonary artery ejection time
PMN: Polymorphonuclear cells
PPE: Pancreatic porcine elastase
SAL: Saline solution
VEGF: Vascular endothelial growth factor
